# Beyond the genome: Public awareness of personalized medicine

**DOI:** 10.34172/hpp.025.43781

**Published:** 2025-12-30

**Authors:** Amir Ghaffarzad, Sepideh Harzand-Jadidi, Nooshin Milanchian, Maryam Mansouri, Amin Talebpour, Ali Mostafaei, Soroush Sharifimoghadam, Hanieh Salehi-Pourmehr, Kavous Shahsavarinia, Sakineh Hajebrahimi

**Affiliations:** ^1^Road Traffic Injury Research Center, Tabriz University of Medical Sciences, Tabriz, Iran; ^2^Emergency and Trauma Care Research Center, Tabriz University of Medical Sciences, Tabriz, Iran; ^3^Department of Internal Medicine, Faculty of Medicine, Tabriz Medical Sciences, Islamic Azad University, Tabriz, Iran; ^4^Student Research Committee, Tabriz University of Medical Sciences, Tabriz, Iran; ^5^Research Center for Evidence-Based Medicine, Faculty of Medicine, Tabriz University of Medical Sciences, Tabriz, Iran; ^6^Urology Department, Faculty of Medicine, Tabriz University of Medical Sciences, Tabriz, Iran

**Keywords:** Personalized medicine, Awareness, Attitudes, Knowledge

## Abstract

**Background:**

Personalized medicine (PM) is becoming increasingly feasible due to advancements in DNA sequencing technology. Our study aims to evaluate the general population’s understanding and attitudes toward PM in Tabriz city.

**Methods:**

In this cross-sectional study, data were collected through a self-administered questionnaire from various participants, including health centers, outpatient cases, and community populations. The research involved forming a panel of specialists, conducting a literature review, developing initial survey questions, seeking expert feedback, revising and refining questions, implementing a pilot study, and finalizing the survey instrument.

**Results:**

The mean (SD) age of 313 participants was 39.63 (14.41) years, with 36.7% being male. About 65% of participants were married, 55.3% were employed, and 79.6% residing in the urban areas. Most participants reported good physical (64.2%) and mental health (58.1%) status. Half of the participants evaluated the different dimensions of their lifestyle as moderate. The majority of respondents had not heard about PM (67.4%; 95% CI: 62.0%-72.4%), and most believed that PM is a promising healthcare approach (61%; 95% CI: 55.5%-66.2%), or can be an essential tool for preventing, diagnosing, and treating various diseases (60.1%; 95% CI: 54.6%-65.3%). Attitudes towards PM were influenced by education level. Economic status influenced knowledge about PM (*P*=0.002). Similarly, attitude toward PM was influenced by economic status (*P*<0.05).

**Conclusion:**

The study revealed that education and economic status significantly influence individuals’ awareness and attitudes towards PM. Education and income levels should be considered while planning for educational programs about PM. Our findings can inform the development of effective strategies for promoting PM in healthcare settings.

## Introduction

 Personalized medicine (PM) refers to tailoring healthcare interventions to an individual’s genetic profile, and it is becoming increasingly feasible due to advances in DNA sequencing technology. This approach offers the potential for more effective diagnosis and treatment.^1-3^

 As PM becomes increasingly feasible due to advances in DNA sequencing, its promise notwithstanding, widespread adoption raises concerns.^4^ It is essential to recognize that while these advancements create opportunities, they also necessitate a robust infrastructure to support their integration into everyday healthcare practices. Continuous innovation in this field is crucial for maintaining momentum in PM adoption.^5^

 A significant barrier to the successful implementation of PM is the general public’s limited understanding of its principles and benefits.^6^ Studies have shown that Americans, including medical students, are not well informed about PM. However, there is limited data on the general population’s perceptions. To fully realize the benefits of PM, it is crucial to address public concerns and ensure that appropriate policies and governance frameworks are in place.^7,8^ This gap in understanding can lead to skepticism and resistance towards new healthcare technologies. Therefore, fostering public awareness through educational initiatives is vital. By informing individuals about how PM works and its potential benefits, we can build trust and encourage acceptance of these innovations. Additionally, exploring public attitudes and perceptions will provide valuable insights into how to communicate the value of PM effectively.^9-11^ It can also determine the next step in developing policies and regulations related to PM.^12^

 Policymakers must appreciate public sentiment to balance moral issues — such as data privacy, access, and fairness — with the potentially beneficial clinical impacts of PM. Furthermore, assessing public interest can guide resource allocation for PM initiatives, ensuring that research and development efforts align with community needs. By prioritizing transparency and public engagement, policymakers can foster an environment conducive to the acceptance and success of PM.^12,13^ A well-informed public is crucial for the successful implementation of PM, as it helps individuals make informed decisions, builds trust in healthcare technologies, and ensures acceptance of PM initiatives.

 This study aimed to assess the extent of public knowledge, awareness, and attitudes toward PM among Tabriz residents in Iran and to identify key sociodemographic predictors of these opinions.

## Methods

###  Study Design 

 This cross-sectional descriptive study aimed to assess the general population’s knowledge, attitudes, and perceptions regarding PM in Tabriz, Iran.

###  Setting and Participants 

 The study was conducted between June 2023 and June 2024 in community and healthcare settings across Tabriz, Iran. Eligible participants were adults ( ≥ 18 years) recruited from healthcare centers, outpatient clinics, and public venues representing diverse socioeconomic districts.

###  Sample Size Estimation

 The sample size was determined using Morgan’s table for sample size calculation, assuming a population size of approximately 1.5–2 million in Tabriz, with a 95% confidence level and a 5% margin of error. According to Morgan’s table, the minimum required sample was 384 participants. However, due to practical constraints, 313 participants were recruited. Although the estimate did not reach the ideal, stratified random sampling across key demographic strata (age, gender, education, socioeconomic status) ensured representativeness and minimized selection bias.

###  Recruitment Procedure

 Participants were recruited from four educational hospitals, affiliated healthcare centers, and community spaces. Data collection venues were selected to reflect socioeconomic diversity across districts.

###  Data Collection

 Data were collected using a structured self-administered questionnaire after obtaining Institutional Review Board approval. Written informed consent was obtained from all participants. Trained research assistants were available to clarify any questions.

###  Survey Development Process

 The questionnaire was developed through a systematic multi-stage process:

- Literature review: Existing surveys on PM identify common themes and knowledge gaps.
- Expert panel consultation: A multidisciplinary panel of 10 experts (clinicians, PM specialists, healthcare administrators, a bioinformatics expert, and an ethicist) guided content development.
- Drafting: Initial questions were formulated covering knowledge, attitudes, and ethical considerations of PM.
- Expert review: The draft was reviewed for clarity, comprehensiveness, and bias minimization.
- Pilot testing: The revised questionnaire was piloted with a small group of healthcare professionals (n≈20) to assess clarity and completion time.
- Finalization: Modifications were made based on pilot feedback, and the final instrument was adopted.

###  Survey Structure

 The questionnaire consisted of three domains: (1) Demographics, (2) awareness of PM, and (3) attitudes toward its application.

###  Measures

####  Demographic Variables

 Demographic data were collected to characterize participants and allow subgroup analyses. Variables included, age (continuous and categorical), gender, marital status, years of education, employment status and occupation, residential location (urban/rural), socioeconomic status (self-rated), media use (television, radio, internet, social media, mobile phone), self-rated physical and mental health, history of chronic conditions, medication use in the past year, history of adverse drug reactions, side effects, or treatment changes due to cost, prior experience with genetic testing or counseling, belief in the role of genetics in health, lifestyle characteristics (exercise, diet, preventive behaviors), and perceived stress level.

####  Awareness Assessment

 Awareness was assessed using multiple-choice and open-ended items that evaluated participants’ familiarity with PM, sources of information, and understanding of ethical implications. A brief definition of PM was provided before these questions.

####  Attitude Assessment

 Attitudes were evaluated using a 5-point Likert scale (1 = strongly disagree to 5 = strongly agree). Items assessed perceptions of the value of PM in prevention, diagnosis, and treatment; willingness to undergo genetic testing; readiness to share genetic data; and concerns regarding cost, privacy, and trust. An open-ended question captured additional reasons for reluctance to adopt PM.

 We have attached the Farsi version of the questionnaire to Supplementary file 1.

###  A Brief Explanation for Participants About Personalized Medicine

 At the beginning of the survey, we used a text to give some information about PM.^8,9,14^

Box 1 provides brief information about PM. Please read below and answer the next questions.

**Box 1. Brief information about personalized medicine**

Today, the treatment for a given disease is somewhat the same in all members of society. While it is not only expensive for everyone to get a prescription, it may have side effects for a group of patients who receive the same treatment prescriptions.

PM is the tendency to take medical measures from population to individualization. That is, instead of using a form of treatment/drug for a given disease in society, according to each person’s personal, genetic, and medical history, treatment of different medicines is considered for them.

PM uses the genetic information of each person in improving the approach and effective methods of prevention, diagnosis, and treatment strategies in chronic diseases, especially diabetes, obesity, osteoporosis, cancers, etc.

###  Statistical Analysis

 Data were analyzed using IBM SPSS Statistics version 21 (IBM Corp., Armonk, NY, USA). Descriptive statistics (frequencies, percentages, means, and standard deviations) were calculated to summarize demographic and survey variables.

 For inferential analyses, associations between categorical variables (e.g., awareness level, attitudes, sociodemographic characteristics) were examined using the chi-square test or Fisher’s exact test when expected cell counts were < 5. Differences in continuous variables between groups were assessed with independent-samples t-tests. All tests were two-tailed, and *P* values < 0.05 were considered statistically significant.

## Results

 Of a total of 850 questionnaire deliveries, only 313 individuals completed the survey, and the others either refused to complete it or filled it out incompletely. The respondents’ mean (SD) age was 39.63 (14.41) (Minimum, maximum: 17.0, 80.0), of which 36.7% were male. Most of the survey participants were married (65.2%) and employed (55.3%). With the majority residing in the urban areas (79.6%). Approximately half of the participants had a moderate economic status (50.2%), and the mean (SD) number of years of education was 32.0 (4.69) (Table 1).

**Table 1:** Baseline characteristics of the participants

**Variable**	**Category**	**Mean±SD**	**N (%)**
Age (y)	–	39.63 ± 14.41	–
Education (y)	–	32.0 ± 4.69	–
Marital status	Single	–	94 (30.0)
	Married	–	204 (65.2)
Sex	Male	–	115 (36.7)
	Female	–	194 (62.0)
Living status	City	–	249 (79.6)
	Urban	–	20 (6.4)
Job status	Unemployed	–	64 (20.4)
	Employee	–	173 (55.3)
	Free job	–	44 (14.1)
Economic status	Poor	–	57 (18.2)
	Moderate or high	–	217 (69.4)
Media use frequency	Radio		
	Very low	–	199 (63.6)
	Low	–	43 (13.7)
	Moderate	–	39 (12.5)
	High	–	14 (4.5)
	Very high	–	8 (2.6)
	TV		
	Very low	–	91 (29.1)
	Low	–	57 (18.2)
	Moderate	–	92 (29.4)
	High	–	60 (19.2)
	Very high	–	9 (2.9)
	Internet		
	Very low	–	47 (15.0)
	Low	–	35 (11.2)
	Moderate	–	75 (24.0)
	High	–	95 (30.4)
	Very high	–	55 (17.6)
Self-reported health status	Physical		
	Very poor	–	7 (2.2)
	Poor	–	23 (7.3)
	Moderate	–	80 (25.6)
	Good	–	137 (43.8)
	Very good	–	64 (20.4)
	Mental		
	Very poor	–	11 (3.5)
	Poor	–	34 (10.9)
	Moderate	–	84 (26.8)
	Good	–	115 (36.7)
	Very good	–	67 (21.4)
Medication use (past year)	Yes	–	105 (33.5)
Changed medication due to:	Ineffectiveness	–	85 (27.2)
	Adverse effects	–	66 (21.1)
	High cost	–	59 (18.8)
Referral to genetic testing/counseling	Yes	–	21 (6.7)

###  Media Use and Health Status

 The reported frequency of media use among the participants varied. Radio and TV were the lowest used media, with 63.6% and 29.1%, respectively. Conversely, 48.0% reported high or very high internet use, while 47.0% reported frequent mobile phone use (Table 1).

 Regarding self-reported physical health, 64 people (20.4%) reported very good health, 137 (43.8%) reported good health, 7 (2.2%) reported very poor health, and 23 (7.3%) reported poor health. In terms of mental health status, 67 people (21.4%) stated that they had a very good status, and 11 people (3.5%) had a very poor or a bad mental health status (10.9%). Seventy-seven participants (24.6%) reported having an underlying disease (Table 1).

 More than half of them did not use any medication in the past year (n = 207).

 Among those who had medication switches, most often arose from ineffectiveness (27.2%), adverse effects (21.1%), or high cost (18.8%). Only 6.7% of respondents had undergone genetic testing or counseling.

 Most respondents believed that genes and genetic structure play a role in physical or mental health (54.6%). 47.6% of the participants rated different dimensions of their lifestyle as average, and others healthy (35.5%). 33.5 percent reported a high or very high daily stress level.

###  Awareness and Attitudes Toward PM

 Only 11.8% of participants had heard sufficiently about PM, and this awareness was obtained via the Internet or articles (16.6%). This percentage clarifies the number of participants who reported familiarity with the concept of PM to a degree that enabled them to articulate its basic principles and significance. Most respondents (61.0%) agreed or strongly agreed with the statement that PM is a promising healthcare approach and can be an essential tool for preventing, diagnosing, and treating various diseases. A total of 41.9% believed that using patients’ genetic information to treat patients is more important than doctors’ clinical experience.

 Half of the individuals (55.3%) believed that using patients’ genetic information in prescribing can improve medication effectiveness, and 52.7% were willing to use their genetic information to improve their treatment. 46.6% indicated willingness to the prescription of different types of drugs or drug doses based on their genetic information. One-third (30.4%) expressed willingness to pay for genetic testing and to reap the benefits of PM (Table 2).

**Table 2:** Awareness and attitudes of participants towardpersonalized medicine

**Questions**	**Very unhealthy **	**Unhealthy **	**Moderate **	**Healthy **	**Very healthy **
How do you evaluate the different dimensions of your lifestyle?	5 (1.6)	24 (7.7)	149 (47.6)	111 (35.5)	22 (7.0)
	**Very low **	**Low**	**Moderate**	**High**	**Very high **
How do you rate your daily stress level?	22 (7.0)	68 (21.7)	116 (37.1)	88 (28.1)	17 (5.4)
	**Yes, enough **	**Not enough **	**No **	**Undecided **	
Have you ever heard of Personalized Medicine?	37 (11.8)	58 (18.5)	211 (67.4)	7 (2.2)	
	**Radio-TV**	**Internet **	**Papers**	**Health centers**	**All of the media **
If the answer to this question is yes, how did you get the information? (n = 97)	26 (8.3)	51 (16.3)	1 (0.3)	17 (5.4)	2 (0.6)
	**Completely disagree **	**Disagree**	**Natural**	**Agree**	**Completely agree **
I believe that Personalized Medicine is a promising healthcare approach.	1 (0.3)	10 (3.2)	99 (31.6)	124 (39.6)	67 (21.4)
I believe that Personalized Medicine can be an essential tool for preventing, diagnosing, and treating various diseases.	-	5 (1.6)	104 (33.2)	123 (39.3)	65 (20.8)
I believe that by using Personalized Medicine, different diseases are better treated.	2 (0.6)	12 (3.8)	117 (37.4)	103 (32.9)	62 (19.8)
I believe that using patients' genetic information to treat them is more critical than physicians' clinical experience.	7 (2.2)	34 (10.9)	121 (38.7)	76 (24.3)	55 (17.6)
I believe that leveraging patients' genetic information in drug administration can improve drug effectiveness.	1 (0.3)	11 (3.5)	112 (35.8)	122 (39.0)	51 (16.3)
I want to use my genetic information to improve my treatment.	-	18 (5.8)	117 (37.4)	99 (31.6)	66 (21.1)
I want to be prescribed different types of drugs or doses based on my genetic information.	1 (0.3)	30 (9.6)	122 (39.0)	93 (29.7)	53 (16.9)
I would be willing to pay for the costs of genetic testing and reap the benefits of Personalized Medicine.	30 (9.6)	58 (18.5)	118 (37.7)	54 (17.3)	41 (13.1)

Data are presented as N (%).

**Figure 1:**
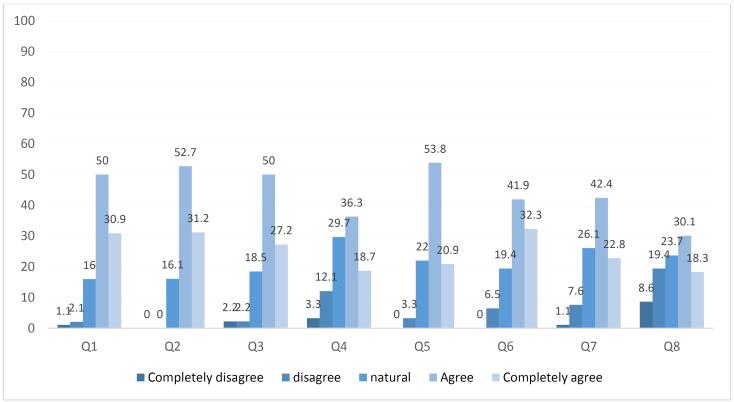
Awareness and attitude of health professionals who had sufficient knowledge about personalized medicine. *Data are presented as a percentage (%). *Note*: Q1. I believe that personalized medicine is a promising healthcare approach; Q2. I believe that personalized medicine can be an important tool for preventing, diagnosing, and treating various diseases; Q3. I believe that by using personalized medicine, different diseases are better treated; Q4. I believe that using patients’ genetic information to treat them is more important than physicians’ clinical experience; Q5. I believe that leveraging patients’ genetic information in drug administration can improve drug effectiveness; Q6. I want to use my genetic information to improve my treatment; Q7. I want to be prescribed different types of drugs or doses based on my genetic information; Q8. I would be willing to pay for the costs of genetic testing and reap the benefits of personalized medicine.

 A reanalysis was conducted among people with sufficient knowledge of PM. The results are illustrated in Figure 1.

###  Associations Between Awareness and Sociodemographic Variables

 We analyzed data across age groups of participants who heard about PM. No significant associations were observed between awareness of PM and age group (*P* = 0.860) or education level (*P* = 0.296). There was a correlation in participants’ economic status as reported by self-reports. Individuals with higher incomes had sufficient (25%) and somewhat sufficient (26.7%) information regarding PM, which was a higher and more significant percentage than in other income groups (Table 3).

**Table 3:** Hearing about PM in different age groups and different economic statuses

	**Yes, enough **	**Yes, not enough **	**No**	***P*** ** value **
Age groups				
17-25 years	7 (13.0)	10 (18.5)	37 (68.5)	0.860^a^
26-35 years	10 (11.8)	16 (18.8)	59 (69.4)	
36-45 years	10 (16.7)	12 (20.0)	38 (63.3)	
46-55 years	4 (7.7)	12 (23.1)	36 (69.2)	
≥ 56 years	6 (12.5)	6 (12.5)	36 (75.0)	
Economic status				
Poor	3 (5.3)	9 (15.8)	45 (78.9)	0.002^a^
moderate	45 (9.9)	29 (19.1)	108 (71.1)	
High	15 (25.0)	16 (26.7)	29 (48.3)	
Education level				
Illiterate or primary	0 (0.0)	1 (6.3)	15 (93.8)	0.211^b^
Secondary and high school	1 (3.1)	8 (25.0)	23 (71.9)	
Diploma	6 (8.2)	14 (19.2)	53 (72.6)	
Academic	18 (14.5)	20 (16.1)	86 (69.4)	

^a^Chi-square; ^b^Fisher’s exact test; Data are presented as N (%).

###  Education, Economic Status, and Attitudinal Differences

 Education and socioeconomic status were noteworthy factors influencing participants’ insights towards PM. Many individuals with a high school diploma or academic degree agreed with this approach, while those with lower levels of education were either opposed to it or indifferent towards it (Table 4). Additionally, the results showed a correlation between individuals’ socioeconomic status and a positive outlook toward the promising approach of PM, with most individuals in the middle- and high-income brackets agreeing or strongly agreeing with this statement, a statistically significant finding.

**Table 4:** Association between awareness and attitude regarding PM based on educational level and economic status

**Variables **	**Completely disagree **	**Disagree**	**Natural**	**Agree**	**Completely agree **	***P*** ** value **	**Variables **	**Completely disagree **	**Disagree**	**Natural**	**Agree**	**Completely agree **	***P*** ** value **
Believing PM is a promising healthcare approach													
**Education level**							**Economic status **						
Illiterate or primary	-	1 (6.3)	10 (62.45)	4 (25.0)	1 (6.3)	**0.003** ^b^	Poor	0 (0.0)	2 (3.6)	30 (54.5)	14 (25.5)	9 (16.4)	**0.003** ^b^
Secondary and high school	-	1 (3.2)	16 (51.6)	9 (29.0)	5 (16.1)		Moderate	1 (0.7)	5 (3.3)	43 (28.3)	60 (39.5)	43 (28.3)	
Diploma	-	4 (5.7)	27 (38.6)	31 (44.3)	8 (11.4)		High	0 (0.0)	2 (3.5)	14 (24.6)	32 (56.1)	9 (15.8)	
Academic	-	3 (2.4)	29 (23.6)	56 (45.5)	35 (28.5)								
Believing PM is a valuable tool for preventing, diagnosing, and treating various diseases													
Illiterate or primary	-	1 (6.3)	10 (62.5)	5 (31.3)	0 (0.0)	**<0.001** ^b^	Poor	-	1 (1.9)	26 (48.1)	17 (31.5)	10 (18.5)	0.054^b^
Secondary and high school	-	1 (3.2)	17 (54.8)	8 (25.8)	5 (16.1)		Moderate	-	3 (2.0)	51 (33.8)	63 (41.7)	34 (22.5)	
Diploma	-	2 (2.9)	29 (42.6)	27 (39.7)	10 (14.7)		High	-	1 (1.8)	10 (18.2)	29 (52.7)	15 (27.3)	
Academic	-	0 (0.0)	30 (24.6)	55 (45.1)	37 (30.3)								
Believing PM is a tool for better treatment of different diseases													
Illiterate or primary	0 (0.0)	1 96.3)	10 (62.5)	3 (18.8)	2 (12.5)	**0.049** ^a^	Poor	0 (0.0)	1 (1.9)	24 (44.4)	14 (25.9)	15 (27.8)	0.159^b^
Secondary and high school	1 (3.3)	1 (3.3)	17 (56.7)	7 (23.3)	4 (13.3)		Moderate	2 (1.3)	8 (5.3)	55 (36.4)	52 (34.4)	34 (22.5)	
Diploma	1 (1.4)	4 (5.8)	32 (46.4)	23 (33.3)	9 (13.0)		High	0 (0.0)	1 (1.8)	16 (29.1)	29 (52.7)	9 (16.4)	
Academic	0 (0.0)	3 (2.5)	37 (30.6)	50 (41.3)	31 (25.6)								
Believing that genetic information to treat is more important than the clinical experience of physicians													
Illiterate or primary	0 (0.0)	1 (6.3)	11 (68.8)	4 (25.0)	0 (0.0)	0.063*	Poor	0 (0.0)	2 (3.7)	31 (57.4)	11 (20.4)	10 (18.5)	0.087^b^
Secondary and high school	0 (0.0)	3 (9.7)	17 (54.8)	4 (12.9)	7 (22.6)		Moderate	3 (2.0)	22 (15.0)	52 (35.4)	41 (27.9)	29 (19.7)	
Diploma	2 (2.9)	10 (14.7)	33 (48.5)	16 (23.5)	7 (10.3)		High	3 (5.4)	5 (8.9)	21 (37.5)	16 (28.6)	11 (19.6)	
Academic	5 (4.2)	16 (13.4)	38 (31.9)	31 (26.1)	29 (24.4)								
Believing that genetic information in drug administration can improve drug effectiveness.													
Illiterate or primary	0 (0.0)	1 (6.3)	10 (62.5)	5 (31.3)	0 (0.0)	**0.002** ^a^	Poor	0 (0.0)	1 (1.8)	26 (47.3)	19 (34.5)	9 (16.4)	0.354^b^
Secondary and high school	0 (0.0)	1 (3.3)	19 (63.3)	6 (20.0)	4 (13.3)		Moderate	0 (0.0)	8 (5.4)	53 (35.6)	61 (40.9)	27 (18.1)	
Diploma	0 (0.0)	4 (5.7)	30 (42.9)	32 (45.7)	4 95.7)		High	1 (0.8)	2 (3.6)	15 (26.8)	27 (48.2)	11 (19.6)	
Academic	1 (0.8)	4 (3.3)	33 (27.3)	53 (43.8)	30 (24.8)								
Willing to use personal genetic information to improve treatment													
Illiterate or primary	-	1 (6.3)	10 (62.5)	4 (25.0)	1 (6.3)	**0.002** ^a^	Poor	-	1 (1.8)	26 (47.3)	13 (23.6)	15 (27.3)	0.083^b^
Secondary and high school	-	5 (16.1)	16 (51.6)	5 (16.1)	5 (16.1)		Moderate	-	11 (7.3)	54 (35.8)	50 (33.1)	36 (23.8)	
Diploma	-	4 95.6)	33 (46.5)	26 (36.6)	8 (11.3)		High	-	3 (5.4)	14 (25.0)	27 (48.2)	12 (21.4)	
Academic	-	5 (4.1)	37 (30.3)	44 (36.1)	36 (29.5)								
Willing to pay for the costs required to do genetic testing and reap the benefits of PM													
Illiterate or primary	2 (12.5)	5 (31.3)	8 950.0)	1 (6.3)	0 (0.0)	0.197^a^	Poor	6 (10.9)	12 (21.8)	29 (52.7)	4 (7.3)	4 (7.3)	0.051^a^
Secondary and high school	2 (6.5)	6 (19.4)	15 (48.4)	3 (9.7)	5 (16.1)		Moderate	17 (11.3)	28 (18.5)	57 (37.7)	30 (19.9)	19 (12.6)	
Diploma	5 (7.0)	12 (16.9)	33 (46.5)	16 (22.5)	5 (7.0)		High	3 95.4)	13 (23.2)	15 (26.8)	14 (25.0)	11 (19.6)	
Academic	14 (11.5)	23 (18.9)	39 (32.0)	25 (20.5)	21 (17.2)								
Willing to prescribe different types of drugs or doses based on genetic information													
Illiterate or primary	0 (0.0)	3 (18.8)	8 (50.0)	5 (31.3)	0 (0.0)	0.119^b^	Poor	0 (0.0)	5 (9.1)	25 (45.5)	16 (29.1)	9 (16.4)	0.956^b^
Secondary and high school	0 (0.0)	3 (9.7)	16 (51.6)	8 (25.8)	4 (12.9)		Moderate	1 (0.7)	13 (8.7)	61 (40.7)	45 (30.0)	30 (20.0)	
Diploma	0 (0.0)	8 (11.3)	38 (53.5)	17 (23.9)	8 (11.3)		High	0 (0.0)	6 (10.7)	19 (33.9)	20 (35.7)	11 (19.6)	
Academic	1 (0.8)	11 (9.1)	38 (31.4)	44 (36.4)	27 (22.3)								

^a^Chi-square; ^b^Fisher’s exact test; Data are presented as N (%).

 Regarding beliefs about the role of PM in the prevention, diagnosis, and treatment of various diseases, individuals with a university education were the largest group to agree with this question, and this difference was statistically significant. Conversely, individuals with high economic status comprised the largest group supporting this option, but the difference was not statistically significant (*P* = 0.054).

 Regarding the question “Believing genetic information in drug administration can improve drug effectiveness” and “Willing to use personal genetic information to improve treatment,” the results indicated that most individuals with secondary and academic education had a favorable opinion, which was statistically significant compared to other educational groups. However, there was no statistically significant difference concerning economic status for these two questions (Table 4).

## Discussion

 This cross-sectional survey assessed awareness and attitudes toward PM among 313 individuals. Overall, participants demonstrated moderate awareness of PM and generally positive attitudes toward its potential for prevention, diagnosis, and individualized treatment. In contrast, direct experience with genetic or pharmacogenomic (PGx) testing remained low. These findings align with several international studies showing a recurring pattern: interest and perceived value of PM are often higher than actual knowledge or prior use of genetic services.^12,15-17^

 Several extensive surveys and reviews have reported similar results. Population studies in Asia and Europe commonly indicate low-to-moderate awareness of PGx and PM, with the internet and media serving as the primary sources of information. Willingness to consider PM frequently exceeds prior personal experience with testing. For instance, a nationwide Korean survey reported that awareness of PGx testing was approximately 28% and participants preferred integrated PGx approaches despite limited prior testing experience, a pattern similar to our participants’ high belief in the role of genetics but low testing uptake.^12^

 Comparable trends have been observed in the United States and Europe: U.S. surveys found generally favorable attitudes toward pharmacogenomic research and testing, though awareness varied across populations and study designs. Similarly, extensive European cross-country surveys identified substantial proportions of respondents lacking sufficient knowledge about PM and genetic testing. Differences between countries are commonly attributed to variability in public education efforts, healthcare infrastructure, insurance coverage, and national genomic medicine policies.^18,19^

 Our findings diverge from some prior reports on the strength of the association between awareness and sociodemographic variables. While many studies have demonstrated strong associations between higher education and greater awareness or acceptance of PM, other surveys, such as those from Korea, reported weaker or inconsistent correlations, particularly when overall awareness was uniformly low. In the present study, education showed a clear positive association with PM knowledge and attitudes, while higher economic status demonstrated a less robust but still significant relationship. This pattern is consistent with numerous international reports identifying education as a dominant predictor of genomic health literacy.^18,20,21^

 Three main factors may explain observed similarities and differences across studies:

1. Information environment and trust: Where public health campaigns, clinician engagement, and media coverage of PM are greater, awareness and acceptance tend to rise. Our participants identified the internet and articles as their primary information sources, a finding echoed in European surveys, underscoring the central role of digital media in shaping PM knowledge.^15^
2. Access and affordability: Cost and availability of PGx testing remain consistent barriers. Many studies have reported a high willingness to undergo testing but a low readiness to pay, mainly due to financial concerns or a lack of reimbursement. This gap helps explain the discrepancy between favorable attitudes and limited real-world testing observed in our sample.^22^
3. Health literacy and education: Educational attainment often predicts comprehension of complex health concepts. Higher education and income levels are typically associated with greater PM knowledge and a more positive attitude toward its implementation, a pattern reproduced in our data. Where some studies have reported weaker associations, limited overall awareness may have reduced their statistical power to detect subgroup differences.^18^

Economic status also influenced participants’ perceptions of PM. Those in higher income brackets reported greater understanding and more favorable attitudes toward PM implementation. This relationship may reflect better access to healthcare resources, information, and services typically available to higher-income individuals. Those with greater financial means may also be more likely to discuss PM through media or healthcare providers.^23^

 Interestingly, while middle- and higher-income participants supported PM, the effect of income was weaker than that of education. This suggests that economic status influences awareness and attitude, but

 Education remains a more decisive factor. The finding highlights the importance of ensuring equitable access to PM across different socioeconomic strata and underscores the need for inclusive educational strategies that reach all segments of society.

 This study provides an essential exploration of public awareness and attitudes toward PM in Iran. Although the sample size was relatively small, the results offer valuable insights for future PM implementation. The findings emphasize the necessity of public education campaigns, professional healthcare training, and policy development to support PM adoption and improve healthcare outcomes in Iran.

 Disparities in PM knowledge are likely influenced by multiple modifiable factors, including (1) general attitudes toward PM and related fields such as genetics and biobanking, (2) lack of awareness about PM research and available care options, (3) varying levels of trust in medical research, and (4) socioeconomic differences. Similar to awareness and attitudes, precision medicine knowledge (PMK) is dynamic and may shape differences in PM uptake. PMK reflects an individual’s health literacy within the context of genomic medicine—specifically, the ability to make informed healthcare decisions, understand genetic influences on health, and appreciate the ethical implications of testing. Enhancing PMK could help reduce disparities in PM engagement. While genomic health literacy remains under-researched, it is essential for participation in PM-related research, interpreting risk assessments, adhering to PM-based medical recommendations, and engaging in policy discussions about data governance. Notably, evidence suggests that differences in PM knowledge and attitudes are more strongly influenced by health literacy than by race or ethnicity.^24-26^

 Several barriers to engagement with genetic testing may offset participants’ optimism about the PM’s potential. Financial constraints remain a key obstacle, particularly for individuals without adequate insurance coverage. This economic limitation can create a gap between participants’ positive beliefs and their actual utilization of genetic services. Cultural factors also play an essential role. Societal norms and cultural perceptions may generate skepticism or misunderstanding about genetic testing, thereby reducing participation. Therefore, culturally sensitive education and communication strategies are essential. Additionally, the perceived complexity of PM may deter engagement; many individuals find genetic concepts difficult to understand. Simplifying these ideas through targeted education could enhance accessibility and participation. Similar patterns have been reported in other countries, including Korea, where public awareness of PGx testing remains low—likely due to differences in healthcare infrastructure, public campaigns, and educational outreach. Understanding these contextual factors can inform strategies to improve engagement with PM.

 Education emerged as a pivotal determinant of PM awareness. Participants with at least a high school diploma were significantly more likely to support PM than those with lower levels of education. This suggests that educational institutions can play a vital role in disseminating information about medical innovations, equipping individuals to make informed health decisions. Higher education is often associated with improved critical thinking and greater access to health information, both of which contribute to more positive attitudes toward PM. Moreover, participants with university degrees were more likely to believe in PM’s potential for disease prevention, diagnosis, and treatment, aligning with prior studies emphasizing the importance of educational initiatives in enhancing understanding of complex medical topics. Healthcare providers and policymakers should therefore prioritize targeted academic programs, particularly for populations with lower educational attainment.^23,27^

## Barriers, Ethical Concerns, and Implementation Implications

 Consistent with international literature, participants identified cost, privacy, and lack of trust or understanding as key barriers to PM adoption. These align with well-documented challenges, such as data-sharing concerns, inadequate clinician education, and policy-level issues related to reimbursement and the national PGx strategy. Overcoming these barriers requires multi-level interventions: public education tailored to low-literacy groups, clinician training in genomic literacy, transparent data-governance frameworks, and financial support mechanisms to prevent the widening of health inequities.^28^

## Strengths and Limitations

 Strengths of this study include the structured questionnaire developed with multidisciplinary expert input, pilot testing, and stratified sampling across multiple healthcare and community settings to enhance representativeness. Limitations include the smaller-than-planned sample size (313 vs the ideal adjusted estimate), potential selection bias inherent to facility-based recruitment (though mitigated through district-level sampling), and reliance on self-reported data, which may be subject to recall or social desirability bias. Therefore, national generalizations should be made cautiously. Additionally, the cross-sectional design precludes causal inference regarding determinants of awareness and attitudes.

## Implications and Future Research

 Our findings among the general population are consistent with our previous research on healthcare professionals, which also revealed limited awareness but positive attitudes toward PM.^29^ Together, these studies indicate a systemic gap in PM knowledge across both the public and provider levels, underscoring the need for coordinated educational and policy strategies to facilitate PM adoption in Iran.

 Future studies should: (1) evaluate the effectiveness of educational campaigns, particularly those targeting low-education and low-income populations, (2) assess clinician readiness and system-level capacity for integrating PGx into clinical practice, and (3) test funding and reimbursement models to improve equitable access. Large-scale, nationally representative surveys and mixed-methods studies could enhance generalizability and provide deeper insight into cultural factors influencing PM acceptance.^30^

## Conclusion

 Education and economic status significantly affect individuals’ awareness and attitudes toward PM. Participants with higher levels of schooling demonstrated greater understanding and more favorable views of PM’s benefits. Similarly, those with higher income levels exhibited greater awareness and positivity toward PM compared with those from lower-income backgrounds. While both factors are essential, education appears to play a more decisive role in shaping PM knowledge. Enhancing education and improving economic accessibility could raise public health literacy regarding PM and reduce disparities in its adoption. Targeted educational and outreach initiatives are essential to empower individuals to engage with PM and foster a more equitable healthcare landscape.

## Competing Interests

 None.

## Data Availability Statement

 Not applicable.

## Ethical Approval

 The regional ethics committee of Tabriz University of Medical Sciences approved this study (IR.TBZMED.REC.1402.843).

## Supplementary Files

Supplementary file 1. Farsi version of the questionnaire
