# Supplementary material for: Beyond the genome: Public awareness of personalized medicine
Source: Health Promot Perspect. 2025 Dec 30;15(4):384–93. doi: 10.34172/hpp.025.43781 (PMC13156256; doi:10.34172/hpp.025.43781)
Supplement: Supplementary file 1 — Farsi version of the questionnaire [file hpp-15-384-s001.pdf]

شماره پرسشنامه: .... تاریخ تکمیل: ....

پرسشنامه ارزیابی آگاهی و نگرش جمعیت عمومی نسبت به پزشکی شخصی سازی شده

با سلام

احتراما به اطلاع شما می‌رساند که پرسشنامه حاضر به منظور اندازه گیری آگاهی و نگرش جمعیت عمومی نسبت به پزشکی شخصی سازی شده تهیه شده است. امید است که با همکاری و تکمیل این پرسشنامه در جهت پیشبرد اهداف پژوهش یاریمان نمایید. لازم به ذکر است که این اطلاعات از لحاظ اخلاقی کاملاً محرمانه بوده و فقط در راستای اجرای این پژوهش استفاده می گردد؛ لذا نیازی به قید نام و نام خانوادگی نمی باشد. خواهشمند است به پرسش‌ها صادقانه پاسخ دهید و هیچ پرسشی را بدون پاسخ نگذارید. پیشاپیش از همکاری شما صمیمانه سپاسگزاریم.

الف) اطلاعات زمینه ای

۱) **سن:** .... سال ۲) **جنسیت:** الف) مرد☐ ب) زن☐

۳) **وضعیت تاهل:** الف) مجرد☐ ب) متاهل ☐ ج) سایر ☐ ۴) **سنوات تحصیلی:** .... سال

۵) **وضعیت اشتغال:** الف) شاغل در بیرون☐ ب) شاغل در خانه☐ ج) بیکار☐ ۶) **محل سکونت:** الف) استان☐ ب) شهر ☐ ج) روستا ☐

۷) وضعیت اجتماعی اقتصادی: ضعیف متوسط خوب

۸) **میزان استفاده شما از رسانه‌های گروهی زیر در طول روز چقدر است؟**

⇦ رادیو: الف) خیلی کم☐ ب) کم ☐ ج) متوسط☐ ج) زیاد☐ د) خیلی زیاد☐

⇦ تلویزیون: الف) خیلی کم☐ ب) کم ☐ ج) متوسط☐ ج) زیاد☐ د) خیلی زیاد☐

⇦ اینترنت: الف) خیلی کم☐ ب) کم ☐ ج) متوسط☐ ج) زیاد☐ د) خیلی زیاد☐

⇦ رایانه: الف) خیلی کم☐ ب) کم ☐ ج) متوسط☐ ج) زیاد☐ د) خیلی زیاد☐

⇦ تلفن همراه: الف) خیلی کم☐ ب) کم ☐ ج) متوسط☐ ج) زیاد☐ د) خیلی زیاد☐

۹) **وضعیت فعلی سلامت جسمی خود را چگونه ارزیابی میکنید؟**

الف) خیلی خوب☐ ب) خوب ☐ ج) متوسط☐ ج) ضعیف☐ د) خیلی ضعیف☐

۱۰) **وضعیت فعلی سلامت روانی خود را چگونه ارزیابی میکنید؟**

الف) خیلی خوب☐ ب) خوب ☐ ج) متوسط☐ ج) ضعیف☐ د) خیلی ضعیف☐

۱۱) **آیا در حال حاضر، به بیماری خاصی مبتلا هستید؟** الف) بلی☐، ب) خیر☐، اگر پاسخ سوال ۱۱ بلی است، نام بیماری را ذکر کنید: ....

۱۲) **آیا در طول یک سال اخیر داروی خاصی مصرف میکنید؟** الف) بلی☐ ب) خیر☐

۱۳) **آیا تاکنون تجربه تغییر دارو به دلیل عدم اثربخشی آن را داشته اید؟** الف) بلی☐ ب) خیر☐

۱۴) **آیا تاکنون تجربه تغییر دارو به دلیل بروز عوارض جانبی ناخواسته را داشته اید؟** الف) بلی☐ ب) خیر☐

۱۵) **آیا تاکنون تجربه تغییر روش درمانی به دلیل هزینه های بالای آن را داشته اید؟** الف) بلی☐ ب) خیر☐

۱۶) **بنظرتان ژن ها و ساختار ژنتیکی تان در وضعیت سلامت جسمی یا روانی شما نقش دارد؟** الف) بلی☐ ب) خیر☐

۱۷) **آیا تاکنون به آزمایش ژنتیک یا مشاور ژنتیک ارجاع داده شده اید؟** الف) بلی☐ ب) خیر☐

۱۸) **ابعاد مختلف سبک زندگی خود (ورزش، تغذیه، پیشگیری از بیماریها، سلامت جسمی، روانی، اجتماعی، اجتناب از مصرف دارو و ...) را چگونه ارزیابی میکنید؟** الف) خیلی سالم☐ ب) سالم☐ ج) متوسط☐ ج) ناسالم☐ د) خیلی ناسالم☐

۱۹) **سطح استرس روازنه خود را چگونه ارزیابی میکنید؟** الف) خیلی پایین☐ ب) پایین☐ ج) متوسط☐ ج) بالا☐ د) خیلی بالا☐

ب) آگاهی

۲۰) **آیا تاکنون در رابطه با پزشکی شخصی سازی شده مطلبی شنیده اید؟**

الف) بلی، درباره آن شنیده ام و اطلاعات دارم ☐

ب) بلی، درباره آن شنیده ام اما اطلاعات خاصی ندارم ☐

ج) خیر، تاکنون درباره آن مطلبی نشنیده ام ☐

- اگر پاسخ سوال فوق بلی است، از چه طریق، اطلاعات کسب کرده اید؟ (میتوانید چندین گزینه را انتخاب نمایید)

الف) رادیو و تلویزیون☐ ب) اینترنت و شبکه های مجازی ☐ ج) روزنامه ها و مجلات☐ ج) مراکز درمانی و کادر درمان☐

د) سایر موارد ☐ (لطفا ذکر شود ....)

*** متن زیر، اطلاعات مختصری از پزشکی شخصی سازی شده را ارائه میدهد. لطفا متن زیر را مطالعه نموده و به سوالات بعدی پاسخ دهید.**

- *امروزه روش درمانی برای یک بیماری معین در همه افراد جامعه تا حدودی یکسان است. در حالیکه پیچیدن یک نسخه درمانی برای همه افراد، نه تنها هزینه اثربخش نیست، بلکه ممکن است عوارض جانبی برای گروهی از بیماران که نسخه های درمانی یکسانی دریافت میکنند، داشته باشد.*
- *پزشکی شخصی سازی شده، گرایش اقدامات پزشکی از جمعیت محور به سمت فرد محور شدن است. بدین معنا که به جای بهره گیری از یک نوع درمان/دارو برای یک بیماری معین در جامعه، با توجه به ویژگی های شخصی، ژنتیکی و سابقه پزشکی هر فرد، درمان/داروی متفاوتی برای وی در نظر گرفته میشود.*
- *پزشکی شخصی سازی شده از اطلاعات ژنتیکی هر فرد در بهبود رویکرد و روش های موثر پیشگیری، تشخیص و استراتژی های درمانی در بیماری های مزمن، به ویژه دیابت، چاقی، استئوپروز، سرطان ها و... استفاده می کند.*

ج) نگرش	کاملاً موافقم	موافقم	نظری ندارم	مخالفم	کاملاً مخالفم
۲۱- من معتقدم که پزشکی شخصی سازی شده، یک رویکرد بهداشتی درمانی امیدوار کننده است.					
۲۲- من معتقدم که پزشکی شخصی سازی شده، میتواند ابزار مهمی برای پیشگیری، تشخیص و درمان بیماری های مختلف باشد.					
۲۳- من معتقدم که با استفاده از پزشکی شخصی سازی شده، بیماری های مختلف، بهتر درمان میشوند.					
۲۴- من معتقدم که استفاده از اطلاعات ژنتیکی بیماران جهت درمان آنها، مهمتر از تجربه بالینی پزشکان است.					
۲۵- من معتقدم که بهره گیری از اطلاعات ژنتیکی بیماران در تجویز دارو میتواند اثربخشی دارو را ارتقا دهد.					
۲۶- من مایلم از اطلاعات ژنتیکی ام برای بهبود روند درمانم استفاده شود.					
۲۷- من مایلم انواع داروها یا دوزهای مختلف دارویی بر اساس اطلاعات ژنتیکی ام تجویز شوند.					
۲۸- من مایلم هزینه های لازم برای انجام آزمایشات ژنتیک و بهره مندی از مزایای پزشکی شخصی سازی شده را بپردازم.					

- در صورتی که مایل به بهره مندی از مزایای پزشکی شخصی سازی شده نیستید، لطفا دلیل خود را ذکر نمایید.

الف) هزینه های بالا ☐

ب) خصوصی بودن اطلاعات ژنتیکی و نگرانی درباره نحوه نگهداری پرونده پزشکی در مراکز درمانی ☐

ج) عدم اطمینان به اثربخشی پزشکی شخصی سازی شده در مقایسه با پزشکی مبتنی بر شواهد ☐

د) سایر دلایل ☐ (لطفا ذکر شود) ....

با تشکر از همکاری شما
